# Analysis of Active and Passive Tobacco Exposures and Blood Pressure in US Children and Adolescents

**DOI:** 10.1001/jamanetworkopen.2020.37936

**Published:** 2021-02-23

**Authors:** Rebecca V. Levy, Kaye E. Brathwaite, Harini Sarathy, Kimberly Reidy, Frederick J. Kaskel, Michal L. Melamed

**Affiliations:** 1 Division of Pediatric Nephrology, Department of Pediatrics, Montefiore Medical Center, Bronx, New York; 2 Division of Nephrology, Department of Medicine, Albert Einstein College of Medicine, Bronx, New York; 3 Division of Nephrology, Department of Medicine, University of California, San Francisco; 4 Department of Epidemiology and Population Health, Albert Einstein College of Medicine, Bronx, New York

## Abstract

**Importance:**

Hypertension is a leading cause of cardiovascular disease in adults; preclinical associations between hypertension and cardiovascular disease are seen in childhood. Nicotine is a known toxin, but its association with pediatric hypertension is unclear.

**Objective:**

To test the hypothesis that tobacco exposure is associated with the presence of elevated blood pressure in US children and adolescents and that this association is dose dependent.

**Design, Setting, and Participants:**

This cross-sectional study used data from the 2007 to 2016 National Health and Nutrition Examination Survey (NHANES), a population-based nationally representative sample of US children and adolescents. Children were eligible if they were aged 8 to 19 years at the time of participation in the main NHANES study. Exclusion criteria included those of the main NHANES study, inability to complete testing, or missing questionnaires. Of the 10 143 participants in NHANES aged 8 to 19 during the study years, 8520 were included in the analysis. Analysis was conducted from October 12, 2019, to July 9, 2020.

**Exposures:**

Tobacco exposure, defined as serum cotinine levels greater than 0.05 µg/L, or reporting living with a smoker or smoking themselves.

**Main Outcomes and Measures:**

Elevated blood pressure, classified as greater than 90% for a child’s age, sex, and height according to the 2017 American Academy of Pediatrics Clinical Practice Guidelines. The a priori hypothesis that there is a positive association between tobacco exposure and elevated blood pressure in the study population was tested. Analysis included logistic regression with adjustment for possible confounders. Subgroup and sensitivity analyses were conducted.

**Results:**

A total of 8520 children were included in the analysis, representing 41 million US children. The mean (SD) age of the participants was 13.1 (0.05) years, 51% (95% CI, 49%-52%) were male, and 58% (95% CI, 54%-62%) were non-Hispanic White individuals. Participants with any tobacco smoke exposure were more likely than those without exposure to be older (mean [SD] age, 13.3 [0.07] years vs 12.8 [0.06] years), male (53% [95% CI, 51%-55%] vs 49% [95% CI, 47%-50%]), and non-Hispanic Black individuals (19% [95% CI, 16%-22%] vs 10% [95% CI, 8%-12%]). The odds of having elevated blood pressure was 1.31 (95% CI, 1.06-1.61) for any tobacco exposure after adjustment; odds were similar across subgroups and remained significant in multiple sensitivity analyses.

**Conclusions and Relevance:**

This study suggests that tobacco exposure is associated with elevated blood pressure in US children and adolescents. This modifiable risk factor represents a target for further research into reducing hypertension in children and adolescents.

## Key Points

**Question:**

Is exposure to tobacco associated with the presence of elevated blood pressure (defined as >90% for age, sex, and height percentiles) in US children and adolescents?

**Findings:**

In this cross-sectional study of 8520 US children and adolescents aged 8 to 19 years, tobacco exposure was associated with statistically significant higher odds of having elevated blood pressure after adjustment for potential confounders.

**Meaning:**

This study suggests that tobacco exposure is a modifiable risk factor associated with the presence of elevated blood pressure in US children and adolescents.

## Teaser

This cross-sectional study uses data from the National Health and Nutrition Examination Survey to test the hypothesis that tobacco exposure is associated with the presence of elevated blood pressure in US children and adolescents and that this association is dose dependent.

## Introduction

Hypertension is a major public health concern affecting 46% of US adults.^1^ Awareness, treatment, and control of hypertension in adults have been increasing over time but remain suboptimal.^2^ Uncontrolled hypertension in adults is associated with a number of major sequelae, including cardiovascular disease (CVD), stroke, and kidney disease. The economic impact of hypertension has been estimated at as much as US $50.3 billion annually.^3^

Unlike adult hypertension, in which cutoff values are defined by associations with clinical outcomes, including CVD, cerebrovascular disease, and chronic kidney disease, pediatric hypertension is defined by statistical cutoff values.^4^ Although pediatric hypertension has not been directly associated with cardiovascular morbidity and mortality, there is a large body of evidence showing that pediatric hypertension is associated with adult hypertension.^5^ Furthermore, pediatric hypertension itself is correlated with subclinical cardiovascular abnormalities, including increased carotid intimal medial thickness^6^ and arterial stiffness.^7^ Pediatric hypertension is an important factor in the life course that may have associations with the burden of adult chronic disease, including CVD, cerebrovascular disease, and chronic kidney disease. Therefore, the need to identify modifiable factors for pediatric hypertension is extremely important.

Tobacco exposure raises blood pressure acutely through sympathetic nervous system activation.^8^ However, literature examining the association of active smoking in adults with chronic hypertension has been mixed.^9,10^ Likewise, a recent meta-analysis of pediatric patients found no association between active or passive smoking and the presence of hypertension.^11^ This finding is surprising given the reported associations of smoking with arterial stiffness in adults^12^ and endothelial dysfunction in children.^13^ Furthermore, these studies were conducted before the update to hypertension guidelines in 2017.^4^ We therefore hypothesized that in a large, representative population of US children and adolescents with homogenous phenotyping of exposure and outcome, tobacco exposure would be associated with elevated blood pressure (EBP).

## Methods

### Study Population

The National Health and Nutrition Examination Survey (NHANES) is a nationally representative, cross-sectional survey of noninstitutionalized, civilian, US citizens conducted continuously in 2-year cycles.^14^ Participants complete an in-depth interview and undergo an examination consisting of medical and physiological measurements as well as laboratory tests. The NHANES uses a complex 4-stage survey design to obtain a representative sample.^14^ Certain groups are intentionally oversampled to increase precision for subgroup estimates; in recent cycles, these groups have included racial/ethnic minority groups at or below 185% of the federal poverty level, participants aged 11 years or younger, or particpants aged 80 years or older.^14^ The NHANES study has been continuously approved by the National Center for Health Statistics Institutional Review Board and Ethics Review Board since 1999. This secondary analysis, using deidentified data, was formally classified as exempt by the Albert Einstein College of Medicine Institutional Review Board. Written informed consent was obtained from all participants older than 12 years and from parent or guardians of pediatric participants of the main NHANES survey, with written child assent from participants aged 7 to 11 years. Specific written consent was not required for this secondary analysis of existing data. This report was drafted in accordance with the Strengthening the Reporting of Observational Studies in Epidemiology (STROBE) reporting guideline for cross-sectional studies.^15^

For this study, we used data on NHANES participants from 2007 to 2016 (the most recent years available). Participants were included in the analysis if they were 8 years or older and younger than 19 years at the time of participation and had completed all relevant testing. Children younger than 8 years were excluded because they did not have blood pressure testing performed as part of NHANES. Blood pressure measurements were also not performed for presence of the following: rashes, gauze dressings, casts, edema, paralysis, tubes, open sores or wounds, withered arms, or arteriovenous shunts were present on both arms or if a blood sample had been obtained from an arm within the last week. Further exclusion criteria were those of the main NHANES study.^14^

### Primary Exposure

Tobacco exposure was considered positive if a participant reported the presence of at least 1 smoker in the home or had a serum cotinine level greater than 0.05 µg/L (to convert to nanomoles per liter, multiply by 5.675). Active smoking was defined as answering yes to the question “During the past 5 days, including today, did you smoke cigarettes, pipes, cigars, little cigars or cigarillos, water pipes, hookahs, or e-cigarettes?” or having a serum cotinine level greater than 10 µg/L. The lower cutoff level (0.05 µg/L), originally determined by the assay limit of detection, remains the standard in recent literature examining adolescent tobacco exposure.^16,17^

Serum cotinine levels were measured by isotope dilution high-performance liquid chromatography and atmospheric pressure chemical ionization tandem mass spectrometry. The lower limit of detection of this assay is 0.015 µg/L; the upper reportable limit is 25 µg/L for a sample volume of 0.2 mL or 400 µg/L for a sample volume of 0.05 mL. The assay has good accuracy, with mean values within 9% of theoretical values at all levels except the lower limit of quantification, where it was within 14% of the theoretical values. It further shows good precision with a within-day coefficient of variation less than 5% at any concentration except the lower limit of quantification, where it was less than 16%.^18^ All samples were analyzed at the Division of Laboratory Sciences, National Center for Environmental Health, Centers for Disease Control and Prevention in Atlanta, Georgia.

### Outcomes

Blood pressure was measured manually on all examinees 8 years or older who met the criteria using a Baumanometer calibrated mercury true gravity wall sphygmomanometer (W.A. Baum). Cuff size was determined based on measurement of the upper arm circumference. All examiners were certified for blood pressure measurement by measurement of blood pressure in 12 volunteers, with 92% of measurements within 2 mm Hg and 100% of measurements within 4 mm Hg. Three consecutive measurements were made after the participant was resting quietly in a sitting position for 5 minutes at the mobile examination center. Measurements were made using the right arm unless specific participant conditions prohibited the use of the right arm, in which case the left arm was used. Measurements were taken 30 seconds apart.

EBP was defined as a mean systolic blood pressure or a mean diastolic blood pressure greater than 90% for age, sex, and height^4^; self-reported history of hypertension (n = 63); or self-reported use of antihypertensive medication (n = 11). Children older than 13 years were considered to have the outcome (EBP) if they had systolic blood pressure greater than 120 mm Hg or diastolic blood pressure greater than 80 mm Hg.

Blood pressure levels equal to or above the 95th percentile for age, sex, and height, or 130/80 mm Hg, were evaluated as a secondary outcome. We have referred to this outcome as “hypertension” throughout for convenience, recognizing that a true diagnosis of pediatric hypertension requires multiple readings at several time points.

### Covariates

Race/ethnicity was self-defined in a written questionnaire. Options were prespecified by the NHANES investigators, with an other including multiracial option. Race/ethnicity was included in the analysis because it is known to be independently associated with hypertension. Body mass index was calculated as weight in kilograms divided by height in meters squared and was categorized by a *z* score for age as normal, overweight (>85%), or obesity (>95%) according to US Centers for Disease Control and Prevention guidelines.^19^ Socioeconomic status was approximated using a family poverty index calculated as the ratio of the participant’s family monthly income to the Department of Health and Human Services poverty cut points specific to family size, year, and state. These ratios were categorized as 130% or less of the poverty threshold, between 130% and 185% of the poverty threshold, or more than 185% of the poverty threshold, in accordance with commonly used cutoff levels for determining eligibility for federal assistance programs. Insurance status was coded as none, private (including also having public insurance), or public only (Medicare, Medicaid, State Children’s Health Insurance Program, military health care, Indian Health Service, state-sponsored health plan, or other government insurance).

### Statistical Analysis

Analysis was conducted from October 12, 2019, to July 9, 2020. Continuous variables were described by mean and SD values if normally distributed and by median values and interquartile ranges otherwise; comparisons between groups were made using the *t* test if normally distributed or using the Mann-Whitney test if not normally distributed. Categorical variables were described as percentages and compared by χ^2^ testing. Participants between the ages of 8 and 18 years who were excluded from the study were analyzed for differences in baseline characteristics compared with participants who were included. Multivariable logistic regression was used to assess the association of the primary outcome, presence of EBP, with the exposure of interest, tobacco smoke exposure. The final model was adjusted for age, sex, race/ethnicity, body mass index *z* score, poverty income ratio category, insurance status, and survey year. A similar analysis was conducted using hypertensive-level blood pressure cutoff values.

Subgroup analyses were conducted by examining age older or younger than 12 years, sex, and race/ethnicity. Sensitivity analyses were conducted using the limit of detection of cotinine as a cutoff for exposure, cotinine as a continuous variable, and smoking status categorized as none (cotinine level <0.05 µg/L and no self-reported exposure) vs passive (cotinine level 0.05-10 µg/L or self-reported household smoke exposure) vs active (cotinine level >10 µg/L or self-reported active smoker). Finally, the association of the odds of EBP with decile of the natural log of cotinine after adjustment for covariates was examined.

Statistical analysis was conducted using Stata, version 15 (StataCorp LLC) with appropriate sampling weights to account for the complex survey design. All *P* values were from 2-sided tests, and results were deemed statistically significant at *P* < .05.

## Results

### Participant Characteristics

**Figure 1.**
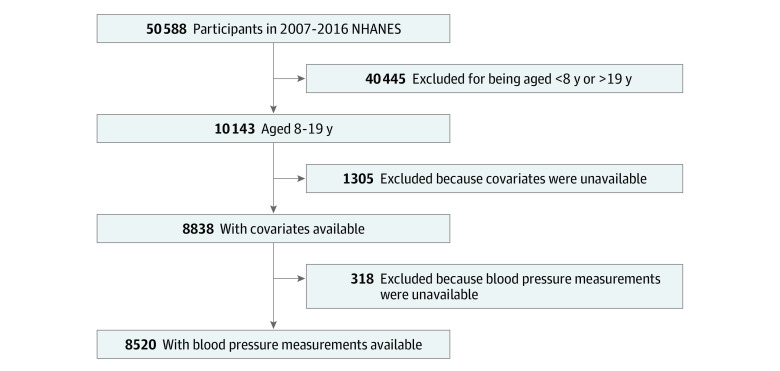
Study Flowchart Flowchart showing study participant selection. Of 50 588 participants in the 2007 to 2016 National Health and Nutrition Examination Survey (NHANES), 8520 remained after fulfilling inclusion and exclusion criteria.

A total of 8520 children from 8 to 19 years of age were included in this analysis, representing 41 million US children. The mean (SD) age at participation was 13.1 (0.05) years, 51% (95% CI, 49%-52%) were male, and 58% (95% CI, 54%-62%) were non-Hispanic White individuals (Table 1). Of the 50 588 participants in the 2007 to 2016 NHANES, there were 10 143 children aged 8 to 19 years; 1305 of these children were missing data on covariates, and 318 children did not have a blood pressure measurement available, leaving 8520 participants in the main analysis (Figure 1). The characteristics of study participants by tobacco exposure status are shown in Table 1. Participants with any tobacco smoke exposure were more likely than those without exposure to be older (mean [SD] age, 13.3 [0.07] years vs 12.8 [0.06] years), to be male (53% [95% CI, 51%-55%] vs 49% [95% CI, 47%-50%]), to be non-Hispanic Black (19% [95% CI, 16%-22%] vs 10% [95% CI, 8%-12%]), to have overweight or obesity (44% [95% CI, 42%-46%] vs 37% [95% CI, 35%-39%]), to have no insurance or only public insurance (59% [95% CI, 55%-62%] vs 36% [95% CI, 32%-39%]), and to have a poverty index less than 130% of the federal poverty threshold (43% [95% CI, 40%-47%], vs 23% [95% CI, 20%-26%]).

**Table 1.** Baseline Characteristics of Study Participants Aged 8 to 19 Years From 2007 to 2016 National Health and Nutrition Examination Survey

Characteristic	% (95% CI)^a^			*P* value
	All (N = 8520)	Tobacco smoke exposure		
		None (n = 4830)	Any (n = 3690)	
Age, mean (SD), y	13.0 (0.05)	12.8 (0.06)	13.3 (0.07)	<.001
Male sex	51 (49-52)	49 (47-50)	53 (51-55)	<.001
Race/ethnicity^b^				
Non-Hispanic				
White	58 (54-62)	57 (53-62)	59 (54-64)	<.001
Black	14 (12-16)	10 (8-12)	19 (16-22)	
Mexican-American	13 (11-16)	16 (13-20)	10 (8-12)	
Other Hispanic	7 (6-9)	8 (7-9)	6 (4-8)	
Other	8 (7-9)	9 (7-11)	7 (6-8)	
BMI category				
Normal	60 (59-62)	63 (61-65)	56 (54-58)	<.001
Overweight	23 (22-24)	22 (21-24)	24 (22-25)	
Obese	17 (16-18)	14 (13-16)	20 (18-22)	
Poverty-income ratio				
<130%	31 (29-34)	23 (20-26)	43 (40-47)	<.001
130%-180%	12 (10-13)	10 (9-12)	12 (10-13)	
>180%	57 (54-60)	67 (63-70)	44 (40-47)	
Insurance				
None	9 (8-10)	8 (7-10)	10 (9-12)	<.001
Private	55 (51-58)	64 (61-68)	41 (38-45)	
Public	37 (34-39)	28 (25-31)	49 (45-52)	
Serum cotinine, median (IQR), µg/L	0.032 (0.011-0.218)	0.011 (0.011-0.023)	0.309 (0.089-1.824)	<.001
Elevated BP^c^	13 (12-14)	11 (10-12)	16 (15-18)	<.001
Hypertension^d^	5 (4-6)	4 (3-5)	6 (5-8)	<.001

Abbreviations: BMI, body mass index (calculated as weight in kilograms divided by height in meters squared); BP, blood pressure; IQR, interquartile range.

SI conversion factor: To convert cotinine to nanomoles per liter, multiply by 5.675.

^a^ Percentages are weighted.

^b^ Self-defined in a written questionnaire; options were prespecified by the National Health and Nutrition Examination Survey investigators, with an “other including multiracial” option.

^c^ Greater than the 90th percentile for age, sex, and height percentile.

^d^ Greater than the 95th percentile for age, sex, and height percentile.

Children who were excluded from the main analysis were more likely than those who were included to be older (mean [SD] age, 13.5 [0.1] years vs 13.0 [0.05] years) and less likely to be White individuals (43% [95% CI, 37%-48%] vs 58% [95% CI, 54%-62%]). Participants not included in the main analysis were less likely to have private insurance than those included (45% [95% CI, 40%-50%] vs 55% [95% CI, 51%-60%]). Differences in sex, body mass index category, and socioeconomic status were not statistically significantly different between missing participants and those included.

### Associations Between Tobacco Exposure and Abnormal Blood Pressure

We tested associations of tobacco exposure with EBP (Table 2). The odds ratio (OR) for the presence of EBP was 1.59 (95% CI, 1.32-1.91) for those with tobacco exposure compared with those without in unadjusted analysis. This ratio was attenuated by adjustment for covariates but remained statistically significant (OR, 1.31 [95% CI, 1.06-1.61]). The OR for the secondary outcome of hypertension was 1.40 (95% CI, 1.05-1.86) after full adjustment.

**Table 2.** Association of Tobacco Exposure With Abnormal Blood Pressure in 8520 Participants of 2007-2016 National Health and Nutrition Examination Survey Aged 8 to 19 Years

Exposure	Elevated blood pressure (>90% for age, sex, and height)			Hypertension (>95% for age, sex, and height)		
	Model 1^a^	Model 2^b^	Model 3^c^	Model 1^a^	Model 2^b^	Model 3^c^
Any tobacco exposure^d^						
OR (95% CI)	1.59 (1.32-1.91)	1.46 (1.21-1.76)	1.31 (1.06-1.61)	1.66 (1.27-2.18)	1.62 (1.23-2.12)	1.40 (1.05-1.86)
*P* value	<.001	<.001	.01	<.001	.001	.02
**Sensitivity analyses**						
Lower cutoff of cotinine (<0.015 µg/L)^d^						
OR (95% CI)	1.61 (1.30-2.00)	1.45 (1.18-1.80)	1.26 (1.01-1.58)	1.64 (1.18-2.30)	1.57 (1.13-2.18)	1.32 (0.95-1.83)
*P* value	<.001	.001	.04	.004	.008	.10
Natural log of cotinine (µg/L)^d^						
OR (95% CI)	1.11 (1.07-1.15)	1.07 (1.03-1.11)	1.05 (1.01-1.09)	1.13 (1.08-1.18)	1.11 (1.07-1.16)	1.08 (1.03-1.14)
*P* value	<.001	<.001	.002	<.001	<.001	.01
Passive tobacco exposure						
OR (95% CI)	1.50 (1.24-1.81)	1.47 (1.21-1.79)	1.33 (1.07-1.65)	1.58 (1.21-2.07)	1.58 (1.19-2.09)	1.37 (1.03-1.83)
*P* value	<.001	<.001	.01	.001	.002	.03
Active smoking						
OR (95% CI)	2.06 (1.46-2.90)	1.41 (1.01-1.96)	1.23 (0.85-1.76)	2.07 (1.32-3.24)	1.81 (1.19-2.78)	1.54 (0.99-2.40)
*P* value	<.001	.046	.26	.002	.002	.06
*P* value for trend	<.001	.001	.03	<.001	<.001	.02

Abbreviation: OR, odds ratio.

SI conversion factor: To convert cotinine to nanomoles per liter, multiply by 5.675.

^a^ Unadjusted.

^b^ Adjusted for age, sex, and race/ethnicity.

^c^ Adjusted for age, sex, race/ethnicity, body mass index category, poverty-income ratio category, insurance status, and survey year.

^d^ Reference category was no tobacco exposure.

#### Subgroup Analysis

**Figure 2.**
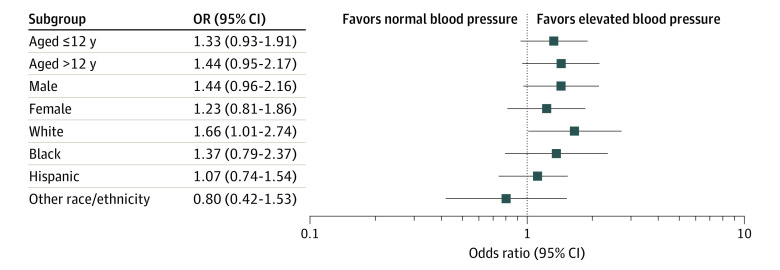
Subgroup Analysis Forest plot showing the association between tobacco exposure and elevated blood pressure for various subgroups of participants aged 8 to 19 years in the 2007 to 2016 National Health and Nutrition Examination Survey. Race/ethnicity was self-defined in a written questionnaire. Options were prespecified by the National Health and Nutrition Examination Survey investigators, with an other including multiracial option.

Analysis of the tobacco-hypertension association stratified by demographic subgroups (age, sex, or race/ethnicity) showed similar ORs within each group (Figure 2). The ORs remained significant for White participants (OR, 1.66 [95% CI, 1.01-2.74]).

#### Sensitivity Analysis

**Figure 3.**
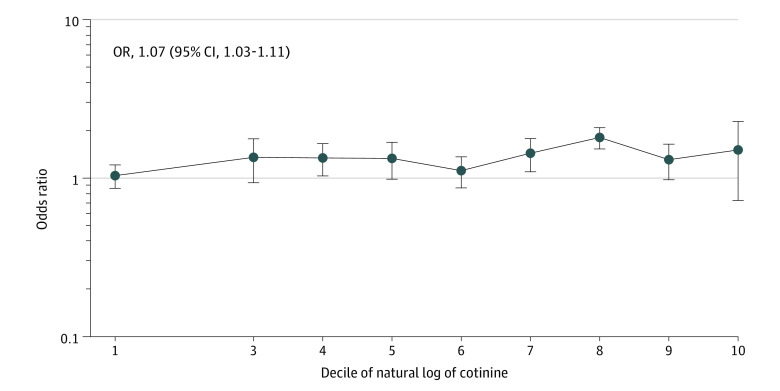
Odds of Elevated Blood Pressure by Decile of Serum Cotinine Level Plot showing odds ratio (OR) of elevated blood pressure by decile of serum cotinine level after adjustment for demographic characteristics, body mass index category, insurance status, socioeconomic status, and survey year. The vertical lines indicate 95% CIs.

Sensitivity analysis with a lower cotinine cutoff (0.015 µg/L) had similar results (OR, 1.26 [95% CI, 1.01-1.58]) (Table 2). Using this threshold classified 69% (95% CI, 66%-72%) of survey participants as exposed. Likewise, using serum cotinine as a continuous variable showed similar results, with a dose-response association between degree of tobacco exposure and blood pressure status. For every increase in the logarithm of cotinine level, the odds of having EBP increased by 5% (95% CI, 1%-9%). Exposure category (no tobacco exposure vs passive exposure vs active smoking) showed a persistent association with EBP in passive smokers (OR, 1.33 [95% CI, 1.07-1.65]). Although statistical significance was lost in the active smoking group (OR, 1.23 [95% CI, 0.85-1.76]), the trend between groups was statistically significant (*P* = .03 for trend) (Table 2). A plot of the OR of EBP by decile of natural logarithm of cotinine is shown in Figure 3.

## Discussion

In this study of 8520 survey participants, representing 41 million US children and adolescents, we found a statistically significant association between tobacco exposure and EBP that persisted after adjustment for demographic characteristics, body mass index, income, and insurance status. The associations were persistent using 2 different definitions of abnormal blood pressure. The association was similar among different participant subgroups defined by age, sex, and race/ethnicity. Finally, sensitivity analyses using different definitions of smoking status showed comparable associations.

### Previous Studies and Biological Plausibility

To date, the literature on the association of tobacco exposure with blood pressure has been mixed, especially in children. Smoking is associated with arterial stiffness in adults^12^ and endothelial dysfunction in children.^13^ However, while some individual studies of children have shown an association of smoking with hypertension,^20,21^ others have not.^22^ A recent meta-analysis of 29 studies including 192 067 children and adolescents found no association between active or passive smoking and the presence of hypertension.^11^ However, the analysis was limited by nonstandard definitions of smoking and significant heterogeneity in study design. Furthermore, these analyses were all conducted prior to publication of the current definitions of pediatric hypertension.^4^ Current guidelines recommend screening children with abnormal blood pressure for personal history of tobacco consumption but have no advice regarding passive tobacco exposure.

Our findings are supported by the existence of data showing a biological plausibility for the association between tobacco exposure and blood pressure. Nicotine causes acute elevations in blood pressure through stimulation of the adrenergic pathway via epinephrine and norepinephrine.^23^ Associations with chronic hypertension are less clear, but a pathway may exist through the development of endothelial damage and vasculopathy. Active smokers have increased arterial stiffness compared with nonsmokers^24^; this finding improves with smoking cessation.^12^ The deleterious association of tobacco exposure with endothelial function is seen even in young children without other risk factors.^13^

### Public Health Impact

The association of tobacco exposure with EBP in children carries important public health implications. An Institute of Medicine report found that raising the legal age to purchase tobacco products to 21 years would result in 249 000 fewer premature deaths and 4.2 million fewer lost life-years among US individuals born during the period from 2010 to 2019.^25^ Because blood pressures in childhood are associated with blood pressures in adulthood and adult hypertension is a risk factor for CVD, modifiable risk factors for pediatric EBP are a potential target for public health campaigns. Although few data on the topic of the indirect costs of hypertension exist,^26^ it is conceivable that a reduction in tobacco exposure among youths may be associated with lower economic and human costs from hypertension and subsequent CVD.

Tobacco use is the second leading risk factor for death in the United States.^27^ With a national adult prevalence of smoking of 15.5%, 5.6 million children can be expected to die prematurely during adulthood from smoking-associated causes.^3^ Smoking is an independent risk factor for CVD and is multiplicative with other risk factors.^28^ Although smoking rates among adults and adolescents have decreased during recent years,^29^ it remains a serious health concern. Furthermore, passive smoke exposure is a risk factor for CVD in adults.^30^ Smoke exposure has unique associations with the life course of children. Maternal smoking is associated with preterm birth.^31^ In children, risks of passive smoke exposure include respiratory illnesses^32^ and CVD in adulthood.^33^ For children with chronic kidney disease, passive tobacco use is associated with greater odds of nephrotic-range proteinuria, which is a risk factor for progression to end-stage kidney disease.^34^ This study supports the hypothesis that the circulatory system in childhood and adolescence is also affected by tobacco in a dose-dependent fashion, as well as the previously known associations already mentioned. Future work may help to elucidate the role of more exact timing and dose duration.

### Limitations and Strengths

Our study had several limitations. As a secondary analysis, we were limited by the inability to collect new data. Thus, there is a risk of residual confounding by unmeasured covariates. Specifically, we were unable to adjust for family history of hypertension, a potentially important confounder, because these data were not collected for children and adolescents. Information on low birth weight, a known risk factor for hypertension, was only available for children younger than 15 years; given the loss of sample size, we chose not to include this covariate to draw conclusions with the most statistical power. Likewise, we had limited information on adolescents who had recently quit smoking, on the duration and timing of passive tobacco exposure, and on tobacco ingestion method (eg, cigarette vs electronic nicotine delivery device vs others).

Nicotine is metabolized in the liver, primarily to cotinine. Cotinine is excreted in the urine and has a longer half-life than nicotine (16 hours vs 2 hours), making it an ideal biomarker for nicotine exposure.^35^ The weaknesses of cotinine as a biomarker include variability in nicotine metabolism, which is known to be associated with genetics, race/ethnicity, sex, kidney function, and concurrent medications.

Although the participants excluded from analysis were more likely to be older, less likely to be White individuals, and more likley to have exclusively public insurance, the included participants were representative of the US population. We were also unable to establish causality in a cross-sectional data set. However, a randomized clinical trial of tobacco exposure is ethically impossible. Stronger observational data would include a prospective, longitudinal study.

This study also has several strengths, including its large size, nationally representative and well-phenotyped population, and inclusion of biomarkers for tobacco exposure. The outcome variable, EBP, was also assessed in a standardized fashion, including replicate measurements to confirm accuracy.

## Conclusions

In this study, tobacco exposure was associated with EBP in US children and adolescents. This association was persistent after adjustment for potential confounders. Sensitivity analyses with alternative exposure and outcome definitions were robust. Thus, tobacco exposure, which is harmful to many body systems, may also be harmful to the cardiovascular system in children and adolescents.
